# Mesenchymal Stromal Cell Proliferation, Gene Expression and Protein Production in Human Platelet-Rich Plasma-Supplemented Media

**DOI:** 10.1371/journal.pone.0104662

**Published:** 2014-08-12

**Authors:** Paola Romina Amable, Marcus Vinicius Telles Teixeira, Rosana Bizon Vieira Carias, José Mauro Granjeiro, Radovan Borojevic

**Affiliations:** 1 Excellion Biomedical Services S.A., Petrópolis, Rio de Janeiro, Brazil; 2 National Institute of Metrology, Quality and Technology, Xerém, Rio de Janeiro, Brazil; National Institutes of Health, United States of America

## Abstract

**Background:**

Platelet-rich plasma (PRP) is increasingly used as a cell culture supplement, in order to reduce the contact of human cells with animal-derived products during *in vitro* expansion. The effect of supplementation changes on cell growth and protein production is not fully characterized.

**Methods:**

Human mesenchymal stromal cells from bone marrow, adipose tissue and Wharton's Jelly were isolated and cultured in PRP-supplemented media. Proliferation, *in vitro* differentiation, expression of cell surface markers, mRNA expression of key genes and protein secretion were quantified.

**Results:**

10% PRP sustained five to tenfold increased cell proliferation as compared to 10% fetal bovine serum. Regarding cell differentiation, PRP reduced adipogenic differentiation and increased calcium deposits in bone marrow and adipose tissue-mesenchymal stromal cells. Wharton's Jelly derived mesenchymal stromal cells secreted higher concentrations of chemokines and growth factors than other mesenchymal stromal cells when cultured in PRP-supplemented media. Bone marrow derived mesenchymal stromal cells secreted higher concentrations of pro-inflammatory and pro-angiogenic proteins. Mesenchymal stromal cells isolated from adipose tissue secreted higher amounts of extracellular matrix components.

**Conclusions:**

Mesenchymal stromal cells purified from different tissues have distinct properties regarding differentiation, angiogenic, inflammatory and matrix remodeling potential when cultured in PRP supplemented media. These abilities should be further characterized in order to choose the best protocols for their therapeutic use.

## Introduction

Human mesenchymal stromal cells (MSC) were first isolated *in vitro* and expanded by Friedenstein and coworkers in 1968 [1], and they were first used in clinical trials by Lazarus and colleagues in the year 1995 [2]. The MSC properties that make them so attractive for regenerative medicine are: 1) they engraft into injured tissues when injected intravenously; 2) they can differentiate into several different cell types; 3) they secrete a wide range of bioactive proteins that stimulate tissue regeneration and inhibit inflammation; and 4) they have immunomodulatory properties [3]. Even when MSC showed promising results *in vitro* studies and in pre-clinical and clinical trials, the mechanism responsible for the obtained results in different pathologies or diseases is not well understood.

MSC are a small cell fraction in primary isolates of mononuclear cells from bone marrow (BM) or adipose tissue (AT), and they have to be *in vitro* expanded when larger quantities of cells are required for a clinical application. Initially, MSC were expanded under standard culture conditions using FBS supplemented media, raising a question of whether adding animal-derived products in the production of cells for human applications was safe. It is known that 100×10^6^ MSC cultivated in FBS-supplemented media carry 7 to 30 mg of bovine proteins that were internalized during the cell expansion period *in vitro* [4]. FBS is not the only animal-derived product that is used during *in vitro* cell expansion: porcine trypsin is used for cell detachment during cell passaging and animal-derived enzymes (like collagenases) are used for initial tissue dissociation. Studies are performed testing potential substitutes, for example, synthetic products or recombinant proteins manufactured under controlled conditions [reviewed by 5] in an attempt to produce clinical-grade cells according to Good Manufacturing Practices. Substitution of tripsin by enzyme-free dissociation methods has shown no detrimental effects on cell viability [6] and the use of trypsin from other origins (corn-derived or recombinat trypsin) resulted in a comparable cell yield, viability and immunophenotype [7]. Supplementation of culture media with human products, such as human plasma [8], [9] or platelet-rich plasma (PRP) [9], have been proposed with success, therefore eliminating the use of FBS.

The question of whether these modifications in cell culture conditions affect MSC pluripotency, engraftment, immunomodulatory and secretory abilities, is still a concern. In the present study, we compared three different cell types: MSC derived from bone marrow, adipose tissue and Wharton's Jelly. We monitored proliferation, cell surface marker expression and adipogenic, osteogenic or chondrogenic differentiation when cells were grown in PRP-supplemented media. We also quantified gene expression and production of cytokines, growth factors and extracellular matrix components into the cell culture supernatants, and we compared cell behavior when cells were cultured in PRP- and in FBS-supplemented media.

## Methods

### Ethics Statements

All donors were informed regarding the study they were participating and they signed an informed consent. The Ethics Research Committee of Pro-Cardíaco Hospital (Rio de Janeiro, Brazil) approved the entire study here described for adipose tissue-derived MSC (AT-MSC; CEP: 55219/12), Wharton's Jelly-derived MSC (WJ-MSC; CEP: 336/10), bone marrow-derived MSC (BM-MSC; CEP: 473/12) and platelet-rich plasma (PRP; CEP: 70649/12).

### PRP preparation

PRP was prepared according to Amable and coworkers [10]. Briefly, blood harvested in ACD-containing tubes (BD, #364606) was centrifuged during 5 minutes at 300 *g*. After separating the platelet-containing plasma above the buffy coat, platelets were concentrated by centrifugation at 700 *g* during 17 minutes and were suspended in a smaller volume of plasma. CaCl_2_ (20 mM) was added in order to initiate platelet activation. An incubation period of 1 hour at 37°C was followed by an overnight incubation step at 4°C. Activated PRP was recovered by centrifugation at 3,000 *g* during 20 minutes and aliquots were frozen at −20°C.

### Cell isolation and culture

Stromal cells were isolated as described before [11]. Briefly, nucleated cells were separated from human bone marrow using Ficoll-Paque PLUS (GE Healthcare, #17-1440-02) by density gradient centrifugation at 700 *g* during 15 minutes. After washing cells with phosphate-buffered saline (PBS - LGC, #13-30259-05), they were plated in T25 flasks in α-MEM (LGC, BR30007-05) supplemented with 10% FBS (LGC, #10-BIO-500) and 10 µg/mL ciprofloxacin (Sigma Aldrich, #17850). Human adipose tissue was washed 3 times with PBS and was treated with 1.76 mg/gr collagenase type I (Sigma, C9891) during 30 minutes at 4°C and 30 minutes at 37°C with agitation. After proteolytic activity inhibition and centrifugation (700 *g*, 7 minutes), pelleted cells were plated in T25 flasks. Human umbilical cord was washed with PBS and blood vessels were removed. Wharton's Jelly was cut into small pieces and digested with 977.4 CDU/gr collagenase type II (Sigma, C6885) at 37°C during 1 hour. Washed cells were centrifuged at 700 *g* during 7 minutes and plated in T25 flasks.

For all the experiments, cells obtained from four different donors in the same passage number were mixed in order to prepare cell pools.

### Proliferation curves

Cells of each cell pool were seeded in 24-well plates at a concentration of 6,000 cells/mL, after being pre-cultured in the corresponding culture media for 5–6 days. α-MEM was supplemented with 10% FBS or different PRP concentrations at the corresponding concentration (1–50%). Every 48 hours, cells grown in different wells were trypsinized and counted in Neubauer haemocytometer.

### Flow cytometry

After cell detachment using a 0.125% trypsin solution, cells were washed with PBS and resuspended in PBS containing 2% FBS. Cell concentration and viability were monitored using Trypan blue in a Neubauer haemocytometer. The following monoclonal antibodies were used as indicated by the manufacturer (BD Pharmingen): CD90-PE (BD, #555596), CD73-FITC (BD, #561254), CD105-FITC (BD,#561443), CD45-FITC (BD,#347463), CD14-PE (BD,#555398), CD34-PEcy5 (BD,#561819), CD31-PE (BD,#555446), IgG-FITC (BD,#555786), HLA-DR-FITC (BD,#555558), CD166-PE (BD,#560903), CD44-PE (BD,#555479), CD54-PEcy5 (BD,#555512), CD146-PE (BD,# 559263). At least 20,000 events were acquired on a BD FACSCalibur flow cytometer and data was analyzed using CellQuest software.

### Differentiation in vitro

Pre-cultured cells were seeded into 24-well plates (1 mL/well) at 26,000 cells/mL (adipogenic differentiation), 10,000 cells/mL (osteogenic differentiation) or cultured as pellets containing 1×10^5^ cells (chondrogenic differentiation). Differentiation medium was changed twice a week.

#### Adipogenic medium

low glucose DMEM (LG-DMEM) supplemented with 10% FBS or 1% PRP, 1 µM dexamethasone (Sigma, D4902), 0.5 mM 3-Isobutyl-1-methylxanthine (Sigma, I7018), 10 µM human insulin (Humulin-N), 0.2 mM indomethacin (Sigma, I7378) and a penicillin/streptomycin solution (LGC, BR30110-01) at 100 U/mL and 100 µg/mL, respectively.

#### Osteogenic differentiation

LG-DMEM supplemented with 10% FBS or 1% PRP, 10 nM dexamethasone (Sigma, D4902), 10 mM β-glycerophosphate (Calbiochem, #35675), 50 µM L-ascorbic acid 2-phosphate (Sigma, A8960) and penicillin/streptomycin at 100 U/mL and 100 µg/mL, respectively.

#### Chondrogenic differentiation

LG-DMEM supplemented with 1% FBS or 1% PRP, 50 µg/mL L-ascorbic acid 2-phosphate, 10 ng/mL transforming growth factor-β3 (Sigma, SRP3171), 0.169 UI/mL human insulin and 6.25 µg/mL human transferrin (Sigma, T8158).

After 17-21 days, cell cultures were fixed in formalin buffer and washed with PBS. Intracellular accumulated lipids were stained with 0.5% Oil Red O solution (Sigma, O0625). Calcium deposits were stained with 1% Alizarin Red S solution (Sigma, A5533), pH 4.2. Glycosaminoglycans were stained with 1% toluidine blue solution (Sigma, #89640).

### RNA extraction and qPCR

RNeasy Plus Mini kit (QIAGEN, #74134) was used for purifying total RNA from pre-cultured (t = 0) and differentiated cells (t = 10 and 21 days). RNA concentration was determined using a Nanodrop 2000 UV-Vis spectrophotometer (Thermo). SuperScript VILO Mastermix (Invitrogen, #11755250) was used for obtaining cDNA from 350 ng RNA in a total reaction volume of 20 µL. A Verity Thermal Cycler (Applied Biosystems) was programmed as follows: 10 minutes at 25°C, 60 minutes at 42°C and 5 minutes at 85°C. qPCR reactions were performed in a Applied Biosystems 7500 Fast Real Time PCR System using TaqMan Gene Expression Mastermix (Applied Biosystems, #4369510), according to manufacturer's instructions. Oligonucleotides and probes for qPCR were purchased from Applied Biosystems (TaqMan gene expression assay, #4331182): *HPRT1* (Hs02800695_m1), *RPL13A* (Hs03043885_g1), *SOX2* (Hs01053049_s1), *POU5F1* (Hs00999634_gH), *TERT* (Hs00972656_m1), *PPARG* (Hs01115513_m1), *ADIPOQ* (Hs00605917_m1), *CEPBA* (Hs00269972_s1), *BMP2* (Hs00154192_m1), *SPARC* (Hs00234160_m1), *RUNX2* (Hs00231692_m1), *SOX9* (Hs00165814_m1), *ACAN* (Hs00153936_m1) and *COL2A1* (Hs00264051_m1).

### Cytokine, growth factor and extracellular matrix quantification

We quantified cell supernatant concentration of 50 different cytokines and growth factors: pro-inflammatory cytokines (GM-CSF, IL-1β, IL-6, IL-8, TNF-α, IFN-γ, IL-2, IL-2R, IL-7, IL-12p40/p70, IL-15 and IL-17), anti-inflammatory cytokines (IL-1RA, IL-4, IL-5, IL-10, IL-13 and IFN-α,), chemokines (eotaxin, IP-10, MCP-1, MIG, MIP-1α, MIP-1β and RANTES), angiogenic factors (VEGF, VEGF-D, endostatin, aFGF, thrombospondin-2, angiopoietin-1, angiogenin and PIGF), matrix metalloproteinases (MMP-1, -3, -7, -8 and -13) and growth factors (EGF, HGF, bFGF, G-CSF, TGF-β1, TGF-β2, TGF-β3, PDGF-AA, PDGF-AB, PDGF-BB and IGF-1). Commercial Luminex kits were used: Human Cytokine 30-plex Assay (Invitrogen, USA), Fluorokine MAP TGF-β Multiplex Kit (R&D, USA), Human Angiogenesis Fluorokine Multi Analyte Profiling Kit (R&D, USA), Fluorokine MAP Human MMP kit (R&D, USA) and Milliplex MAP Human IGF-1 Single Plex Kit (Millipore, USA). Only PDGF-AB was quantified using an ELISA kit: Quantikine hPDGF-AB ELISA (R&D, USA). Procedures were performed according to manufacturer's instructions.

Extracellular matrix proteins were also quantified in supernatants using commercial ELISA kits and following manufacturer's instructions: heparan sulfate (E0623h, EIAab, China), aggrecan (E91908Hu, USCN, USA), decorin (E92127Hu, USCN, USA), elastin (E91337Hu, USCN, USA), laminin (E90082Hu, USCN, USA), perlecan (E82748Hu, USCN, USA), fibronectin (E90037Hu, USCN, USA) and collagens I (E90571Hu, USCN, USA), II (E90572Hu, USCN, USA), III (E90176Hu, USCN, USA), IV (E90180Hu, USCN, USA).

We analyzed supernatants obtained from proliferation experiments at day 8, at the end of the exponential growth phase. PRP pools and control supernatants were also quantified. Supernatant concentration was subtracted by the corresponding control concentration in order to identify protein secretion or consumption, reported as positive or negative values, respectively. In order to make results comparable, concentrations were normalized by cell concentration and culture time, therefore expressing them in pg/10^6^ cells/day.

## Results and Discussion

### PRP yield and recovery

Six different PRP pools were prepared from blood of at least 3 different donors in each, obtaining a final PRP volume of 176.0 mL (7.5±2.8% of the total initial blood volume). Platelets were concentrated 11.9±4.1 times, recovering 104.7±48.6% of the initial total platelet amount. Initial and final platelet concentrations were 2.1±0.8 and 29.4±19.0 × 10^5^ platelets/µL.

All proteins quantified in cell culture supernatants (cytokines, chemokines, growth factors, angiogenic factors, MMPs and extracellular matrix components) were also quantified in PRP pools. Values obtained were within the limits determined previously for PRP samples (data not shown), confirming the reproducibility of the method [10].

### Proliferation of mesenchymal stromal cells under PRP supplementation

Cells were thawed and pre-cultured for up to 6 days with different supplement concentration: 1, 2.5, 5, 10, 20, 30, 40 and 50% PRP and 10% FBS. It took 4 days to the cells in order to adapt to the new supplement concentration (data not shown); pre-culturing cells under the corresponding condition allowed us to study proliferation rate with cells already adapted to their new culture media, since it took at least 2–3 days for cells to exchange internalized proteins from the previous culture media [4]. Cells cultured in media with 40 and 50% PRP died at the end of the pre-culture period, indicating possible PRP inhibitory effects at such high concentrations.

**Figure 1:**
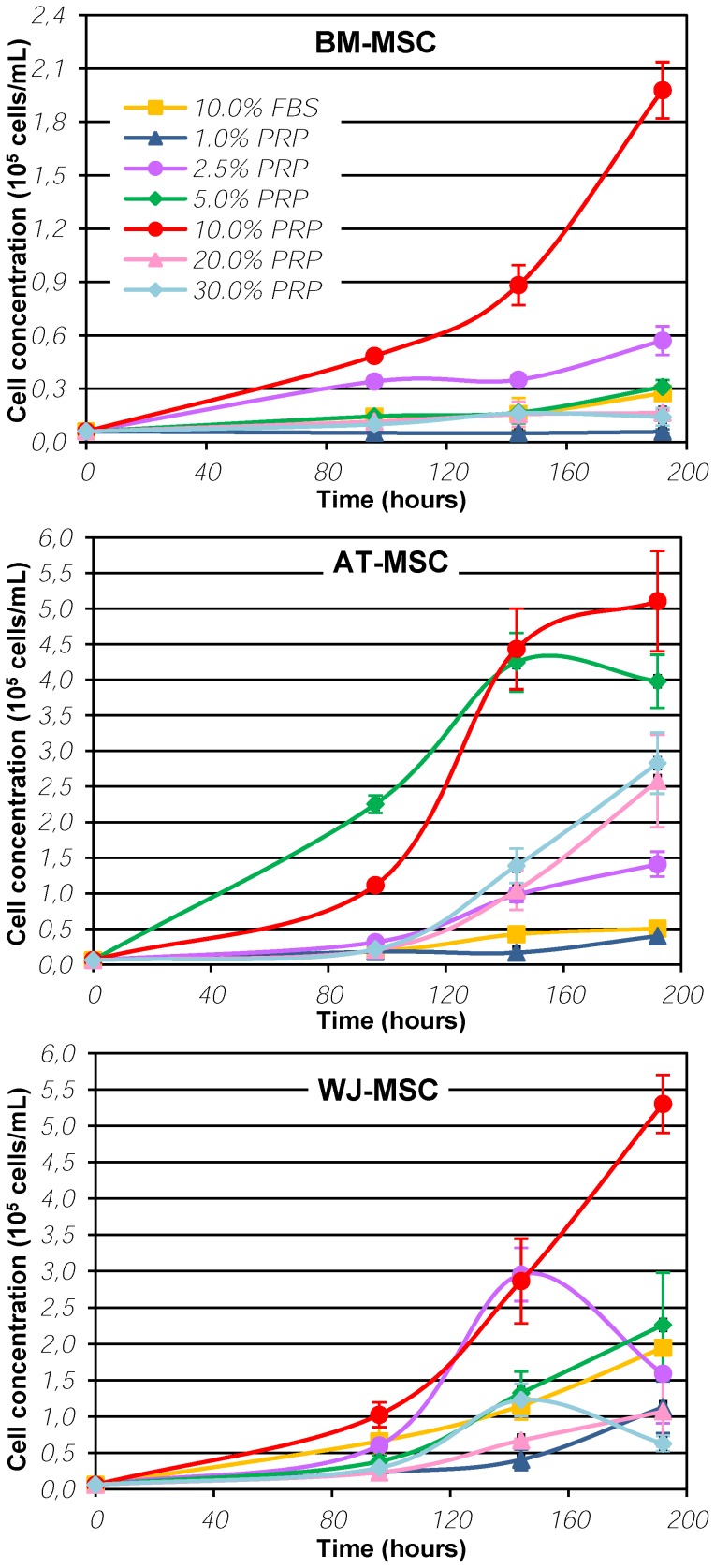
Proliferation curves of human bone marrow- (BM-), adipose tissue- (AT-) and Wharton's Jelly-derived mesenchymal stromal (WJ-MSC) in α-MEM supplemented with human platelet-rich plasma (PRP) in concentrations from 1 to 30% and compared with the proliferation in media supplemented with 10% fetal bovine serum (FBS).

Proliferation curves are shown in Figure 1 and cumulative proliferation times can be found in the Table 1. For all cell types, 10% PRP sustained the highest proliferation rate and the shortest population doubling time. PRP concentrations higher than 10% inhibited cell growth. Cho and coworkers, who compared 10 and 30% PRP as cell culture supplements during 12 days, already reported similar results [12]. Bernardo and colleagues found that BM-MSC are better grown in 10% platelet lysate when compared to lower PRP concentrations, but they have not tested higher concentrations [13].

**Table 1:** Cumulative population doubling time (expressed in mean hour ± standard deviation) for human bone marrow- (BM-), adipose tissue- (AT-) and Wharton's Jelly-mesenchymal stromal cells (WJ-MSC) when grown in α-MEM supplemented with human platelet-rich plasma (PRP) at different concentrations and compared to values obtained when the same cells were cultured in α-MEM supplemented with 10% fetal bovine serum (FBS).

	hours	10% FBS	1% PRP	2.5% PRP	5% PRP	10% PRP	20% PRP	30% PRP
**BM-MSC**	96	79.5±16.1	145.7±74.46	38.3±0.3	79.9±18.4	31.9±0.3	103.6±17.9	111.9±3.1
	144	79.7±2.1	154.3±14.6	56.6±2.4	82.7±4.2	37.3±1.7	84.2±0.6	102.3±12.5
	192	88.4±8.1	185.2±48.6	59.5±3.8	81.7±6.9	38.1±0.8	126.9±1.3	146.5±49.2
**AT-MSC**	96	59.8±3.3	58.3±8.0	38.3±1.4	18.4±0.3	22.8±0.1	52.6±5.5	48.9±3.2
	144	51.5±3.4	96.0±3.8	35.9±1.3	23.5±0.6	23.2±0.7	35.4±3.2	32.0±1.9
	192	63.5±6.6	71.0±5.5	42.3±1.6	31.8±0.7	30.0±1.0	35.7±2.6	34.7±1.4
**WJ-MSC**	96	28.4±3.6	50.5±6.9	28.9±1.2	36.6±4.3	23.6±1.3	49.6±3.2	42.0±1.5
	144	34.0±1.8	54.4±9.1	25.7±0.9	32.6±2.7	26.0±1.5	41.4±0.6	33.3±2.2
	192	38.4±1.7	46.2±4.4	38.3±2.0	37.3±3.9	29.7±0.5	48.1±7.6	57.1±4.1

For AT-MSC, cells grown in 5 and 10% PRP showed a similar proliferation rate until 180 hours, but 5% PRP was not able to support further growth, while 10% PRP maintained high proliferation rates for a longer time (data not shown). Growth in 10% FBS was similar to that in 1% PRP for AT-MSC, population doubling times being 59.8±3.3 and 58.3±8.0 hours in 10% FBS and 1% PRP, respectively. WJ-MSC cultivated in 10% FBS had a similar proliferation rate to the cells grown in 2.5% PRP-supplemented medium, and the same was observed for BM-MSC supplemented with 5% PRP. BM-MSC population doubling times were 79.5±16.1 hours in 10% FBS and 79.9±18.4 hours in 5% PRP. For BM- and WJ-MSC, 10% PRP was clearly the best supplement concentration that promoted cell expansion, with duplication times of 31.9±0.3 and 23.6±1.3 hours, respectively, at the 4^th^ day.

Only a few studies report the effects of different PRP concentrations on cells and when such analyses were done we cannot compare the results obtained because data regarding PRP preparation and quality are lacking. Coincidently or not, the best PRP concentration for all the three cell lines in the present study was 10%. In our PRP preparations, the platelets concentrated approximately by 10 times, meaning that hypothetical final platelet concentration in our cell culture media would be close to the physiological concentrations (if platelets would be still intact after PRP preparation). It would be very valuable to determine the same relation for other studies, using different PRP preparation protocols and different cell lines. We should be able to confirm that physiological concentration of the PRP content is the optimal one, information that we still do not have, even with clinical trials in course for a variety of applications.

### Expression of cell surface markers

Cell cultures in medium supplemented with 10% PRP were analyzed regarding cell surface markers expression by flow cytometry. Results are shown in the Table 2. All three MSC showed values expected for MSC: more than 90% cells were positive for CD73, CD90 and CD105, and less than 5% were positive for CD14, CD34 and CD45. Similar results have been already described [13], [14].

**Table 2:** Quantification of cell surface markers by flow cytometry (expressed in mean percentage ± standard deviation) in human adipose tissue- (AT-), bone marrow- (BM-), and Wharton's Jelly-mesenchymal stromal cells (WJ-MSC) when grown in α-MEM supplemented with 10% human platelet-rich plasma (PRP).

Marker	BM-MSC	AT-MSC	WJ-MSC
**CD73**	96.9±4.7	99.5±0.6	87.2±14.7
**CD90**	94.7±9.0	99.6±0.1	99.7±0.2
**CD105**	98.9±1.2	98.0±1.5	91.1±7.8
**CD45**	2.7±2.4	1.1±1.3	2.0±1.3
**CD14**	1.3±0.9	1.1±1.3	0.9±0.6
**CD34**	1.3±1.1	0.4±0.8	3.0±3.6
**CD31**	0.7±0.3	0.9±0.8	3.6±4.8
**CD44**	99.9±0.1	99.6±0.1	96.4±4.3
**CD54**	47.7±18.7	9.5±8.3	96.7±0.6
**CD146**	88.9±5.7	19.3±7.0	91.7±3.6
**CD166**	88.2±19.9	92.6±9.5	94.1±4.4
**HLA-DR**	0.6±0.7	0.9±1.1	2.6±2.9
**IgG**	0.1	0.0	0.1

When comparing results obtained here with previously reported results for the same cells cultured in 10% FBS-supplemented media, no differences can be found [15]. Cell surface marker expression was similar for all the cell types, except for CD54 and CD146. CD54 was expressed in 47.7±18.7% BM-MSC, less than 10% AT-MSC (9.5±8.3%) and more than 95% WJ-MSC (96.7±0.6%). Studies in mesenchymal cells obtained from the dermis correlated CD54 expression with higher proliferation and higher differentiation potential into adipocytes, osteoblasts and chondrocytes [16]. On the other side, CD146 expression was higher for WJ-MSC (91.7±3.6%) and BM-MSC (88.9±5.7%) and lower in AT-MSC (19.3±7.0%). CD146 was suggested to be a marker of multipotency by Russell and coworkers in 2010, since they found that tripotent clones expressed 2-times higher CD146 cell surface amounts than unipotent clones [17]. Similar results regarding CD54 and CD146 expression were obtained when cells were grown in media supplemented with 10% FBS [15].

### Adipogenic, osteogenic and chondrogenic differentiation

**Figure 2:**
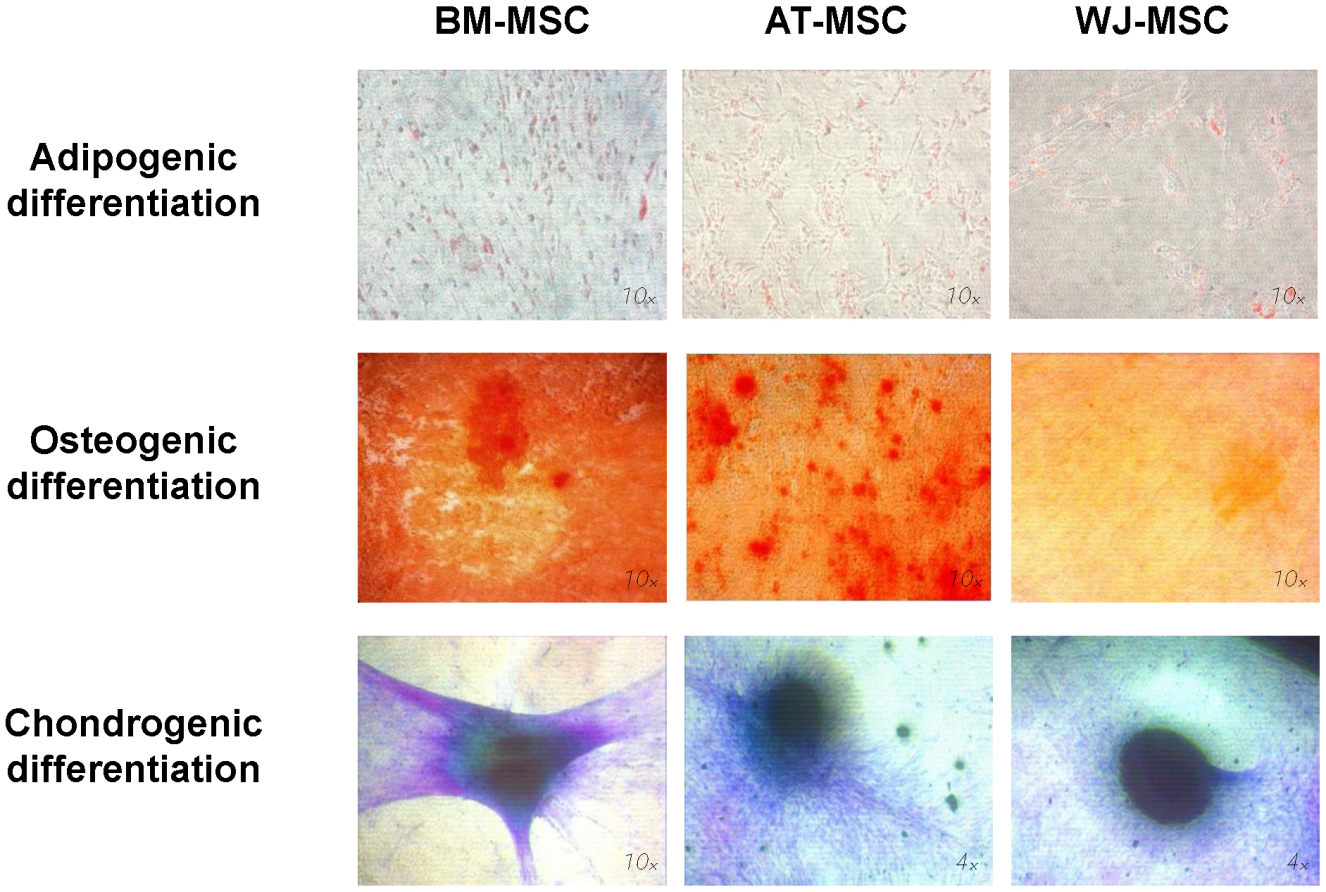
Cell differentiation assays of human bone marrow- (BM-), adipose tissue- (AT-) and Wharton's Jelly-derived mesenchymal stromal (WJ-MSC) into adipogenic, osteogenic and chondrogenic phenotypes under 10% human platelet-rich plasma (PRP) supplemented medium.

Cells pre-cultured in α-MEM supplemented with 10% PRP were differentiated into adipocyte-, osteoblast- and chondrocyte-like cells for up to 21 days (Figure 2). Differentiation controls were run in parallel for all conditions and they were all negative (data not shown).

None of the three MSC proliferated well in adipogenic medium supplemented with PRP, as observed in the Figure 2. Adipocyte-like cells generated from AT- and WJ-MSC in PRP showed small lipid vacuoles with low lipid accumulation, suggesting they were in an earlier differentiation stage or that adipogenesis was impaired. This is more evident if we compare the present results with those obtained when the same cells were differentiated in 10% FBS-supplemented media [15]: cell density for BM-MSC adipogenically differentiated under FBS and PRP was 51,750 and 26,000 cells/mL, respectively; bigger lipid vacuoles were observed for all cells under FBS supplementation.

For AT-MSC and BM-MSC, calcium accumulation was remarkably increased when PRP was used as a culture supplement, but no changes were observed for WJ-MSC. No reports about PRP effect on calcium accumulation were found in the literature for WJ-MSC, but similar results were already described for AT- and BM-MSC. Calcium deposits were increased in BM-MSC *in vitro* when PRP was supplemented in culture media [18]. It has been also reported that AT-MSC plus PRP have a higher osteogenic potential than AT-MSC alone for *in vivo* bone regeneration in a critical size defect in sheep tibia [19].

Under chondrogenic differentiation conditions, differences were less pronounced for all the three MSC. Judging by pellet sizes, AT- and WJ-MSC were more chondrogenic than BM-MSC, since pellets were bigger under PRP-supplemented cultures. No differences between FBS- and PRP-cultured cells could be observed regarding proteoglycans staining and pellet size.

For all cell types, PRP reduced adipogenic capacity and increased osteogenic and chondrogenic differentiation.

### Gene expression

Gene expression of pluripotent (*SOX2*, *POU5F1* and *TERT*), adipogenic (*PPARG*, *ADIPOQ* and *CEPBA*), osteogenic (*BMP2*, *SPARC* and *RUNX2*) and chondrogenic (*SOX9*, *ACAN* and *COL2A1*) markers were studied, evaluating PRP effects on gene expression. Appropriate reference genes were *RPL13A* for WJ- and AT-MSC and *HPRT1* for BM-MSC [11].

*TERT* expression was not detected in any sample. Published data are contradictory regarding *TERT* expression: some authors described *TERT* mRNA as a pluripotent marker in BM-MSC [20] but many others detected neither *TERT* mRNA [21] nor TERT activity [22], [23] in MSC primary cultures.

PRP effects on mRNA expression are shown in the Table 3. Negative values mean downregulation of mRNA expression by PRP, when compared to FBS-cultured cells. PRP increased expression of pluripotent genes in AT-MSC but differentiation markers were all down-regulated, except for the *BMP2* osteogenic marker. *COL2A1* was not detected in any sample. PRP slightly upregulated pluripotent and adipogenic markers in BM-MSC but differences are not significant; expression of osteogenic marker RUNX2 was reduced and *BMP2* expression was upregulated. For chondrogenic markers, PRP reduced *ACAN* expression. When correlating expression results with differentiation assays, mRNA expression of adipogenic markers was reduced in AT-MSC and so was lipid accumulation, when comparing PRP- against FBS-supplemented media. Calcium deposits were increased in both BM- and AT-MSC but only *BMP2* mRNA was upregulated under PRP supplementation.

**Table 3:** Effect of PRP supplementation on gene expression (expressed in mean ± standard deviation as the ratio between expression in PRP cultures and FBS cultures) for human adipose tissue- (AT-), bone marrow- (BM-) and Wharton's Jelly-mesenchymal stromal cells (WJ-MSC) when grown in α-MEM supplemented with 10% human platelet-rich plasma (PRP).

		BM-MSC	AT-MSC	WJ-MSC
**pluripotent genes**	**SOX2**	1.3±0.2	2.7±0.9	(−1.7)±0.5
	**POU5F1**	1.0±0.1	1.2±0.2	1.5±0.6
**adipogenic markers**	**PPARG**	1.2±0.5	(−1.7)±0.1	2.1±0.7
	**ADIPOQ**	1.6±1.5	(−2.8)±0.2	*
	**CEBPA**	1.9±0.3	(−1.4)±0.2	3.0±1.0
**osteogenic markers**	**BMP2**	2.2±0.3	13.2±5.2	1.1±0.3
	**SPARC**	(−1.3)±0.1	(−4.5)±0.0	(−11.8)±0.1
	**RUNX2**	(−2.7)±0.2	(−1.4)±0.2	1.3±0.3
**chondrogenic markers**	**SOX9**	1.2±0.2	(−1.5)±0.3	1.1±0.2
	**ACAN**	(−9.0)±0.1	(−3.6)±0.1	(−3.7)±0.2
	**COL2A1**	**	***	***

*not detected in PRP sample;

**not detected in FBS sample;

***not detected in any sample.

Negative values mean downregulation on mRNA expression by PRP, when compared to FBS-cultured cells, and positive values, upregulation.

In WJ-MSC, *ADIPOQ* was only detected when cells were cultured in FBS-supplemented medium, but PRP induced *PPARG* and *CEBPA* mRNA expression. Regarding osteogenic markers, PRP downregulated significantly *SPARC* mRNA, with minor or no effect on *RUNX2* and *BMP2* mRNA expression. Regarding chondrogenic markers, *SOX9* was not affected by PRP and *ACAN* mRNA expression was reduced.

*COL2A1* was not detected in any FBS-derived sample and it was only detected in PRP-cultured BM-MSC. *ACAN* was downregulated in all cell types. Previous studies reported that pellet technique was not efficient in upregulating *ACAN* expression, especially in AT-MSC when compared to BM-MSC [24].

We detected only insignificant changes in *SOX9* expression in WJ- and BM-MSC after 7 days, even when proteoglycans were detected by toluidine blue staining. This could be due to a faster regulation of *SOX9* expression in the first hours after chondrogenic induction, as demonstrated by Spreafico and coworkers [25].

### Protein secretion

All the 61 analytes were quantified in PRP pools and results were similar to those already reported [10]. New angiongenic factors found to be activated by PRP were: angiogenin (<250.0 pg/mL), thrombospondin (9.8±0.7 ng/mL), PlGF (41.0±4.3 pg/mL), aFGF (479.1±84.7 pg/mL), VEGF-D (359.2±139.9 pg/mL), endostatin (41.5±3.2 ng/mL) and angiopoietin-1 (129.5±28.9 ng/mL). Extracellular matrix protein concentrations in activated PRP were: 202.1±71.6 ng/mL perlecan, <1.25 ng/mL decorin, >150.0 µg/mL heparan sulfate, 216.2±7.4 µg/mL fibronectin, 833.1±719.7 ng/mL aggrecan, 166.2±54.1 ng/mL elastin, 386.5±152.4 ng/mL laminin, 322.2±193.6 ng/mL collagen I, 8.0±6.8 ng/mL collagen II, 33.2±44.0 ng/mL collagen III and 23.8±13.6 ng/mL collagen IV.

Results from cell supernatant quantifications in PRP-supplemented cultures are shown in Table 4. Among all chemokines quantified, only MIG, RANTES and MIP-1α were not detected in any supernatant. RANTES was secreted only by AT-MSC and WJ-MSC in FBS-supplemented media [15]. AT-MSC secreted MIP-1β in detectable concentrations under PRP supplementation; this chemokine was not secreted when the same cells were grown in FBS-supplemented medium, suggesting that PRP induced its secretion. Comparing all the cell lines, AT-MSC secreted lower chemokine concentrations, suggesting a more anti-inflammatory profile. When comparing cell behavior in both culture media, WJ-MSC showed a reduced chemokine secretion, while AT-MSC and BM-MSC increased it, thus concluding that PRP can induce a pro- or anti-inflammatory profile, depending upon the mesenchymal cell origin.

**Table 4:** Cytokine, growth factor and extracellular matrix protein concentration (expressed in mean pg/10^6^ cells/day ± standard deviation) in human adipose tissue- (AT-), bone marrow- (BM-) and Wharton's Jelly-mesenchymal stromal cells (WJ-MSC) supernatants when grown in α-MEM supplemented with 10% human platelet-rich plasma (PRP). nd: not determined (concentration below the detection limit).

pg/10^6^ cells/day	BM-MSC	AT-MSC	WJ-MSC
**Chemokines**			
**Eotaxin**	0.1±0.0	9.0±0.7	2.9±0.4
**IP-10**	1.0±0.3	0.7±0.1	1.1±0.1
**MIP-1β**	2.5±0.2	0.5±1.1	0.7±0.0
**MCP-1**	1,917.3±327.2	1,717.2±206.1	5,840.6±966.8
**Pro-inflammatory cytokines**			
**IL-2R**	18.6±1.9	7.8±1.8	4.7±0.9
**IL-6**	920.1±173.6	272.7±18.0	1,031.5±180.6
**IL-7**	62.7±13.0	21.1±3.4	15.6±2.8
**IL-8**	776.8±217.7	922.8±72.5	5285.1±1261.9
**IL-12**	39.2±15.5	6.9±3.8	3.9±1.4
**IL-15**	11.7±6.5	nd	0.7±1.1
**Anti-inflammatory cytokines**			
**IFN-α**	18.3±3.4	17.1±2.3	10.0±2.6
**IL-1ra**	77.4±16.3	59.3±6.3	103.3±20.1
**Angiogenic factors**			
**Angiogenin**	nd	5,915.3±1,849.7	1,981.4±1,786.1
**Thrombospondin**	3,286.2±330.6	2,481.2±111.2	13,857.8±1,674.1
**PIGF**	3.4±0.4	1.6±0.5	0.5±0.2
**aFGF**	6.2±3.9	0.2±1.2	2.4±2.9
**VEGF-D**	16.2±2.2	8.9±0.0	5.0±4.1
**Endostatin**	nd	239.8±135.1	444.6±58.6
**Angiopoietin-1**	nd	nd	774.8±299.3
**VEGF**	993.1±187.9	494.7±46.6	nd
**Growth factors**			
**TGF-β1**	73.2±523.7	354.2±178.2	1,195.4±273.4
**TGF-β2**	12.1±2.2	0.4±0.8	15.4±1.2
**HGF**	144.5±27.7	163.4±19.0	1,127.0±272.3
**G-CSF**	3.8±2.4	10.4±12.8	65.7±44.1
**Extracellular matrix proteins**			
**Collagen I**	2.6±2.3	84.7±10.5	2.5±1.2
**Collagen II**	nd	2499.4±1625.9	nd
**Collagen III**	42,254.3±3,0542.6	6,980.7±2,352.8	47,336.2±2,685.6
**Collagen IV**	nd	3,186.3±201.4	nd
**Elastin**	5,275.0±1,484.5	5,360.8±2,209.5	4,215.9±491.9
**Fibronectin**	nd	734.4±240.4	119.6±29.6
**Heparan sulfate**	3102.0±2275.4	3,935.1±1,732.7	2128.1±345.9
**Laminin**	3615.5±2971.1	2308.0±1240.1	2021.3±625.8
**Aggrecan**	nd	10.2±1.0	nd
**Matrix metalloproteinases**			
**MMP1**	2,519.2±816.4	22,460.7±3,821.6	2,113.9±366.2
**MMP3**	45.6±4.2	4,056.6±353.1	180.7±75.3
**MMP7**	70.2±60.0	10.8±37.3	23.2±8.3
**MMP8**	69.8±0.0	nd	nd
**MMP13**	179.3±467.1	nd	nd

GM-CSF, TNF-α, IL-1β, IFN-γ, IL-2 and IL-17 were not detected in any supernatant. IFN-γ and IL-1β were only detected in BM-MSC supernatant when cultured in PRP-supplemented media (0.60±0.40 and 1.18±0.72 pg/10^6^ cells/day, respectively). IL-15 was only secreted by BM-MSC and WJ-MSC in PRP-supplemented media. PRP only increased pro-inflammatory profile of BM-MSC, when comparing the results previously obtained for FBS-supplemented cultures with those obtained here; also BM-MSC secreted the highest concentration of 4 of the 6 pro-inflammatory cytokines quantified (IL-2R, IL-7, IL-12 and IL-15).

Only IL-1RA and IFN-α were detected among all the anti-inflammatory cytokines quantified. IFN-α was not secreted by AT-MSC in FBS-supplemented media in detectable concentrations, but PRP induced its expression. WJ-MSC secreted high amounts of IL-1RA, but PRP supplementation reduced its secretion. Therefore, in FBS-supplemented medium, WJ-MSC showed an anti-inflammatory profile, followed by BM-MSC. PRP treatment improved the anti-inflammatory behavior only in AT-MSC.

All angiogenic factors were quantified. In PRP-supplemented cultures, BM-MSC did not secrete angiogenin, endostatin and angiopoietin-1 in detectable concentrations. VEGF and angiopoietin-1 were not detected in WJ- and AT-MSC, respectively. BM-MSC showed a pro-angiogenic profile, secreting the highest concentrations of VEGF, PLGF, aFGF and VEGF-D. AT-MSC was the less angiogenic factor-secreting cell, and PRP reduced secretion for all the cell lines evaluated, when compared with secretion in FBS-suplemented cultures [15].

EGF, bFGF, TGF-β3, PDGF-AA, -AB and –BB and IGF-1 were not detected in any supernatant, meaning that concentrations were lower than the detection limit of the corresponding assays. PDGF-BB was secreted only by BM-MSC in FBS-supplemented medium, showing that PRP downregulated secretion of these 2 growth factors. When comparing with values obtained in FBS supernatants, PRP increased HGF secretion of all cell types. WJ-MSC secreted the highest concentration of all 4 growth factors (TGF-β1, -β2, HGF and G-CSF) in PRP-supplemented cultures. G-CSF was secreted only by WJ-MSC under FBS supplementation but PRP induced G-CSF secretion in all cell lines. Considering the whole growth factor family here quantified, WJ-MSC showed the highest mitogenic profile, followed by AT-MSC. The PRP reduced growth factor secretion for all cells.

With the exception of perlecan and decorin, all extracellular matrix proteins were also detected. AT-MSC secreted the highest concentrations of collagen I and II, elastin, fibronectin, heparin sulfate and aggrecan. FBS-supplemented AT-MSC secreted the highest concentration of collagen III but PRP increased its secretion by BM- and WJ-MSC. BM-MSC secreted the highest amounts of elastin and laminin. Aggrecan was only secreted by PRP-cultured AT-MSC; also, fibronectin was not detected in BM-MSC and WJ-MSC did not secrete any detectable concentration of collagens type II an IV.

All matrix metalloproteinases were detected. MMP1 (collagenase 1) and MMP3 (stromelysin 1) were secreted in the highest concentration by WJ-MSC. BM-MSC secreted the highest amounts of MMP7 (matrilysin); MMP8 (collagenase 2) and MMP13 (collagenase 3) were not detected in AT- and WJ-MSC supernatants. When compared with secretion in FBS-supplemented cultures, PRP increased secretion of MMP for all cell lines. This effect was also observed for MMP1 and MMP3 in PRP-supplemented cultures of human synovial fibroblast; the authors suggested that this catabolic profile should be further studied, specially for cartilage applications [26].

The use of MSC both in autologous and allogeneic therapies may require extensive *in vitro* cell expansion. While FBS is a standard and reference complement of culture media, its substitution is required in order to avoid cell contact with compounds of animal origin, and their transfer to cells that will be used in human therapies. The use of human platelet-derived stimuli for cell expansion in culture has been already proposed, as well as the use of PRP as liquid or jellified carrier for MSC introduction into the tissue that requires repair and regeneration [27]–[29]. In both cases, a full characterization of MSC response to a well defined PRP preparation is required, and the present study gives a contribution to this area.

## Conclusions

PRP increased proliferation rate for all the three cell types. Maximum effects were observed when PRP was supplemented in a 10% concentration, and no changes were observed regarding expression of surface markers. PRP increased calcium deposits and reduced lipid accumulation in AT-MSC and BM-MSC. Regarding gene expression, PRP upregulated pluripotent marker mRNA expression in AT-MSC and BM-MSC, downregulated adipogenic marker expression in AT-MSC, and increased *BMP2* mRNA expression in all three MSC.

Considering protein quantification in supernatants, BM-MSC showed a higher pro-inflammatory and angiogenic behavior, WJ-MSC showed a higher mitogenic profile and AT-MSC were the best producers of extracellular matrix components. PRP reduced mitogenic and pro-angiogenic profile and increased extracellular matrix protein secretion in all the cell types studied.
